# Patient Travel Experiences for Methadone Dosing Visits

**DOI:** 10.1001/jamanetworkopen.2026.37601

**Published:** 2026-10-06

**Authors:** Elizabeth J. Austin, Varuna Ravi, Alexander J. Gojic, Elenore P. Bhatraju, James Darnton, Paul Grekin, Sean Soth, Steve Woolworth, Kevin A. Hallgren, Judith I. Tsui, Emily C. Williams

**Affiliations:** 1 Division of General Internal Medicine, Department of Medicine, School of Medicine, University of Washington, Seattle; 2 Department of Health Systems and Population Health, School of Public Health, University of Washington, Seattle; 3 Evergreen Treatment Services, Seattle, Washington; 4 Department of Psychiatry and Behavioral Sciences, School of Medicine, University of Washington, Seattle; 5 Health Services Research & Development Center of Innovation for Veteran-Centered and Value-Driven Care, Veterans Affairs Puget Sound Health Care System, Seattle, Washington

## Abstract

**Importance:**

Patients often experience travel-related barriers to methadone dosing at opioid treatment programs (OTPs), but the nature of their experiences are not well understood.

**Objective:**

To characterize patient experiences with travel to OTPs for regular methadone dosing visits at a large multisite OTP in Washington state.

**Design, Setting, and Participants:**

This qualitative study utilized an embedded mixed-methods design. Our sample included patients currently receiving methadone treatment at 2 OTP sites in Washington state from November 2024 to February 2026 (including mobile units that operated as extension of the sites). Data were analyzed between February and July 2026.

**Exposures:**

Experiences of travel to OTPs for regular, in-person observed methadone dosing.

**Main Outcomes and Measures:**

Results of a survey administered to patients regarding the modality, time, and cost (eg, gas, childcare) of traveling to the OTP. A subsample of surveyed patients were interviewed about their experiences with and perspectives on traveling for dosing visits. Survey data were analyzed descriptively. Interviews were coded using rapid assessment process.

**Results:**

Our sample included 98 survey participants and 16 qualitative interview participants (median [IQR] age, 43.0 [38.8-50.3] years; 8 cisgender women [50.0%]). Among survey respondents, 54 (55.1%) drove themselves to the OTP, 21 (21.4%) were driven by another person, 12 (12.3%) took public transportation, and 6 (6.1%) took insurer-sponsored medical transportation; 18 (18.4%) reported a disabling condition and 16 (16.2%) needed an assistive device for walking. Participants spent a mean (range) 82.5 (10.0-330.0) minutes round trip and $15.80 ($0-$200.00) in travel-related costs for each visit. Qualitative interviews illuminated that, while distance was often a barrier, unpredictable OTP wait times or conflicts with work, medical, or family obligations also mattered considerably. Participants shared that dosing visits directly prohibited their ability to work, but missing visits put participants at risk for return to opioid use and treatment stagnation.

**Conclusions and Relevance:**

In this mixed-methods qualitative study, requiring regular visits for in-person observed methadone dosing presented significant structural barriers that affect patients’ treatment experience, adherence, and lives. Increasing the flexibility and accessibility of methadone treatment—through policies and novel care practices that actively seek to minimize burden of travel to the OTP and destigmatize care—is essential to enhance care quality and equity.

## Key Points

**Question:**

What are patient experiences with travel to opioid treatment programs for methadone dosing visits?

**Findings:**

This qualitative study found that patients spent a mean 82.5 minutes round trip and $15.80 in travel-related costs for each dosing visit and experienced multiple structural barriers beyond travel distance alone that stagnated progress toward treatment and life goals.

**Meaning:**

These results suggest that requiring regular visits for in-person observed methadone dosing presents major challenges for patients, but increasing the flexibility and accessibility of methadone treatment may enhance care quality and equity.

## Teaser

This qualitative study of US adults receiving methadone treatment summarizes and analyzes experiences of patients who travel to receive treatment, highlighting stressors associated with travel.

## Introduction

Opioid use disorder (OUD) continues to be a significant public health problem resulting in substantial morbidity and mortality.^1^ Approximately 4% of the US adult population have OUD; yet their access to first-line treatment—like methadone—continues to lag.^2^ Data from 2022 showed that only 25% of people with OUD received medications for OUD (MOUD).^3^ In the US, methadone can only be dispensed at a certified opioid treatment program (OTP) and has strict guidelines that require regular (often daily) attendance to ensure doses are directly observed.^1^ Pandemic-era federal policy changes now allow for more rapid increase of take-home doses, or doses that patients can take outside the OTP (also referred to as *carries*).^4^ Despite the recognition that increasing flexibility offers benefits,^5,6^ the interpretation and implementation of these policies varies widely.^7,8,9^

Patients often experience travel-related barriers to attending methadone dosing visits.^10,11,12,13,14,15^ For example, studies have found that that patients spend 37 to 45 minutes driving to their OTP, as compared with 15 minutes to their nearest primary care clinic.^14,16^ Travel burden increases significantly for patients who take public transportation, and 26% of patients may not be able to access a methadone clinic within 180 minutes of travel time.^16,17,18^ Consequently, transportation is the most commonly cited barrier to retention in methadone treatment.^12^

Travel distance alone may not provide the full picture of accessibility and barriers to in-person dosing at OTPs.^16^ Other factors like cost, unanticipated wait times at the OTP, or the physical labor of travel (eg, walking, standing) also influence patient perceptions of accessibility and travel burden, but are obscured in measures that reflect distance alone.^16^ Illuminating and elevating these experiences may help identify how OTPs can increase methadone accessibility. Therefore, we conducted a mixed-methods evaluation of patient experiences with travel to OTPs for dosing visits.

## Methods

### Study Setting

This study took place in 2 sites affiliated with a large nonprofit OTP in Washington state that serves a large catchment area (approximately 6 counties, 9000 square miles). The OTP has clinical protocols to begin considering take-home doses as early as the first few weeks of treatment, based on assessments of patient safety and dose stability. This study occurred in the context of a larger study to implement a mobile health (mHealth) tool for dosing directly observed by video, aiming to expand take-home doses for patients with attendance barriers, including but not limited to transportation barriers.^19,20^

### Study Design

We utilized an embedded mixed methods design (rooted in pragmatism),^21^ where quantitative survey and qualitative interview data were gathered simultaneously (with qualitative data considered primary)^22^ and connected to provide convergence and complementarity.^23^ Study activities were approved by the University of Washington Institutional Review Board (No. STUDY00011142). Reporting follows guidelines from the American Association for Public Opinion Research (AAPOR) and Standards for Reporting Qualitative Research (SRQR) reporting guidelines.^24,25^

### Sample

Our nonprobability sample included patients receiving methadone at either of the 2 sites (including mobile units that operated as extension of the sites). To be eligible, patients needed to be enrolled in treatment for at least 30 days (meaning they completed steps for their treatment onboarding and initial dose stabilization) and enrolled in the video dosing program (meaning they were eligible for take-home doses).

### Data Collection

From November 2024 to February 2026, we invited all video dosing program patients to complete a brief survey about their transportation experiences. The survey was verbally administered by OTP or research staff and recorded electronically. The survey asked participants to self-report their travel modality, estimated average time, distance, and cost (including direct costs, eg, gas or transit fare, and indirect costs, eg, childcare or lost earnings from work) for round trip travel to the OTP for a regular dosing visit, and their disability status. While many participants were traveling to the OTP less frequently due to video dosing program enrollment, the logistics of travel they reported on the survey (time, distance, cost) had not changed.

From January to December 2025, we recruited a subsample of surveyed patients for qualitative interviews. Interview participants completed a verbal informed consent process that included a discussion of how study data would be used. Interviews asked open-ended questions about their experiences getting to the OTP, factors that influenced their ability to attend dosing visits as planned (prior to the video dosing program), and their perspectives on effects from travel. Interviews also asked participants to self-report their racial and ethnic identity (using US census-defined categories) and other demographics characteristics known to be associated with health care access and related disparities. Interviews followed a discussion guide developed by a multidisciplinary team of researchers and clinicians and were conducted by phone by 2 researchers (E.J.A. and V.R.) with qualitative experience. Interview participants received a $50 gift card.

### Statistical Analysis

Survey data were cleaned; free-text responses were reviewed by members of the study team and normalized (eg, converting participant responses to be the same unit of measurement) via discussion and consensus. Survey data were analyzed descriptively. Data were analyzed with Excel version 16.110.3 (Microsoft Corp).

#### Qualitative Analysis

Interview recordings were professionally transcribed and coded using a combination of deductive and inductive approaches.^22^ First, all transcripts were read and coded using rapid assessment process, where coders used structured coding templates to summarize and reduce data to neutral (eg, influencing factors that could have a positive or negative valence) domains.^26,27^ Next, coders engaged in inductive coding of quotes to identify themes. Coders iteratively reviewed coding with other members of the research team to get feedback on data interpretations. The team then reviewed quantitative and qualitative data together to enhance interpretations of both data sources and themes.^28^

## Results

Our sample included 98 survey participants and 16 interview participants. Among interview participants, median (IQR) age was 43.0 (38.8-50.3) years; 8 (50.0%) identified as cisgender women; 14 (77.7%) identified as White and 3 (18.8%) as having Latino ethnicity (Table 1). Among the full survey respondent cohort, 54 (55.1%) drove themselves to the OTP, 21 (21.4%) were driven by another person, 12 (12.3%) took public transportation, and 6 (6.1%) took insurer-sponsored medical transportation; 18 participants (18.4%) reported a disabling condition and 16 (16.2%) reported using an assistive device for walking. Participants reported spending a mean (range) 82.5 (10-330) minutes round trip and $15.80 ($0-$200) in travel-related costs (eg, gas, transit fare, lost wages, childcare) per visit (Table 2). Four themes emerged that characterize travel experiences and barriers (Table 3).

**Table 1.** Interview Participant Characteristics^a^

Characteristics	No. (%) (N = 16)^b^
Age, median (IQR), y	43 (11.5)
Gender identity	
Woman (cisgender)	8 (50.0)
Man (cisgender)	7 (43.8)
Woman (transgender)	0
Man (transgender)	0
Nonbinary, genderqueer, gender nonconforming	1 (5.6)
Race	
American Indian or Alaskan Native	2 (11.1)
Asian	1 (5.6)
Black or African American	0
Native Hawaiian or other Pacific Islander	0
White	14 (77.7)
Other (Puerto Rican, Indigenous)	1 (5.6)
Ethnicity	
Hispanic or Latino	3 (18.8)
Not Hispanic or Latino	13 (81.3)
Primary language	
English	16 (100.0)
Other	0
Highest level of education completed	
Middle school or less	0
Some high school, no diploma	3 (18.8)
High school diploma or GED	4 (25.0)
Some education after high school, did not receive degree or certificate	4 (25.0)
Associate’s degree or vocational certificate	5 (31.3)
4-y College degree (BA/BS), Master’s degree, or other terminal degree	0
Housing status	
Own or rent	11 (68.8)
Staying in single-room occupancy	0
Staying with family member	2 (11.1)
Staying with friend or nonrelative	1 (5.6)
Residential addiction program	0
Shelter	0
On the street without shelter	1 (5.6)
Other (Section 8 housing)	1 (5.6)

Abbreviation: GED, General Educational Development.

^a^ All responses were self-reported.

^b^ Percentages are rounded and may not sum to 100.

**Table 2.** Participant-Reported Characteristics of Travel to the Opioid Treatment Programs (OTP) for Dosing Visits

Characteristics	Participants, No. (%)^a^
**Average time and cost of travel^b^**	
Time spent traveling round trip for dosing visit, min	
Mean (range)	82.5 (10-330)
Median (IQR)	60 (30)
Distance traveled 1-way for dosing visit, miles	
Mean (range)	20.3 (1-80)
Median (IQR)	15 (21)
Total cost round trip for single dosing visit (direct and indirect costs incurred), $	
Mean (range)	15.80 (0-200)
Median (IQR)	10.00 (15.00)
**Travel modality and participant disability status**	
Mode of transportation	
Drive self	54 (55.1)
Driven by another	21 (21.4)
Public transportation	12 (12.3)
Medical transportation (eg, Medicaid transportation service)	6 (6.1)
Taxi service	1 (1.0)
Walk	1 (1.0)
Bike	1 (1.0)
Multiple modalities and/or may vary by day	2 (2.0)
Disability that makes travel difficult?	
Yes	18 (18.4)
No	78 (79.6)
Did not know or refuses to answer	2 (2.0)
Use of assistive device^c^	
Cane	11 (11.2)
Walker	2 (2.0)
Seeing-eye dog	1 (1.0)
Wheelchair	2 (2.0)
None	83 (84.7)
Missing	1 (1.0)

^a^ Percentages are rounded and may not sum to 100.

^b^ Some responses were missing, incomplete, or otherwise could not be included in the average: time spent had 9 excluded responses, distance traveled had 5 excluded response, and total cost had 6 excluded responses.

^c^ Options not mutually exclusive.

**Table 3.** Summary of Themes and Example Quotes

Theme	Description	Representative participant quotes (participant identifier)
Getting to the OTP varied in modality and burden, and could be unpredictable	Travel time varied, and was sometimes long (ranging 10-330 min per dosing visit) Modalities for getting to OTP were unpredictable and changed over time (only 55.1% drove themselves) Need for alternate modalities to be ready Participants with disabilities (18.4%) experienced difficulty traveling	“I used to ride the bus, and that was horrible. I hated doing that. But now my partner drives me if I need to go in, and that’s only every two weeks.” (Participant 2) “Sometimes [medical transportation] don’t show up. So, I do have to find a ride.” (Participant 10)
OTP hours, arrival policies, and wait times made attending dosing visits harder	OTP operating hours felt limiting Policies about arrival times posed a barrier to getting take-home doses Wait times for dosing hard to predict and often long	“The only downfall is that they close really early. So, if you’re not really a morning person, you always feel like you’re running late going there.” (Participant 7) “If I have to do it one day a week, it’s not a problem, you know. However 5 days a week, 6 days a week, it wasn’t gonna make sense.” (Participant 1)
Regular dosing visits were often at odds with life obligations, like employment or other medical care	Cost for travel and missed work felt burdensome (ranging from $0-$200 per visit) Life obligations (health, family, work) sometimes took precedence over dosing visits Seeing other people at the OTP who reminded participants of drug use or encouraged their drug use	“You have other important areas in your life that you need to focus on too, and that’s usually the case with people in treatment.… Or any given thing because I know single moms that might be their reason, or if there’s just any other thing that you have a lot of time or that you need [focus on].” (Participant 11) “It makes me really uncomfortable when I go into the clinic and I feel like it’s being run by the addicts basically.” (Participant 2)
Barriers to attending dosing visits contributed to return to use and stagnated treatment and recovery progress	Missing dosing visits left participants feeling physically unwell To manage symptoms, some participants used drugs, which influenced their well-being, progress toward recovery, and treatment	“When I had to go in 3 or 4 times a week, I would miss doses periodically because I couldn’t get in, or I couldn’t get away from an appointment or something that I had that day that ran over, something like that. And it made things a little more difficult for me because missing 1 dose isn’t that big a deal necessarily because it’s still in your system. But you do feel it at the end of the night. You’re not that comfortable, and your symptoms can come back if you miss more than a day. And I’ve done that before, and it was pretty horrible.” (Participant 2)

Abbreviation: OTP, opioid treatment program.

### Theme 1: Travel to the OTP 

Participants described that time spent traveling to the OTP varied widely and could be unpredictable. Some participants found travel to be easy, noting that the OTP site was “pretty close, it’s conveniently located [and] a really centralized location.” Others described much longer commutes to get to the OTP, which felt burdensome. As one participant explained, “So, 6 days a week, I was in my car driving to [OTP location] and back. It’s like a little over an hour, hour and a half round trip every day, 6 days a week. I did have a gas card that I was getting from the state to help with gas and stuff, but it was still a pain in the butt.”

Participants shared that sometimes the hardest part about traveling to the OTP was figuring out how they were going to get there, not how much time it would take. Inclement weather, delays or no-shows from public transportation or medical transportation for people with physical disabilities or changing schedules often threw travel plans into a lurch. Modalities also changed over time as life circumstances changed, such as gaining or losing access to a car or dependable ride.

“Right now, I have a car. So, I drive myself,” explained a participant. “Before, when I first started going, I didn’t have a car. So, the friend that I was just talking about, she actually used to drive me every day, which was really hard because having to go every day when you don’t have a vehicle.” In general, participants felt that, in the words of one, “it was just really hard to get to and from there” for regular dosing visits.

### Theme 2: OTP Hours, Arrival Policies, and Wait Times

Participants shared that several factors related to how the OTP operated made attending dosing visits more difficult, beyond the time it took them to travel there. For example, participants shared that OTP operating hours (typically ranging from early morning [5:30-6:30 am] to early afternoon [12:30-2:00 pm]) felt limiting: “I wish they were open later, though.… They’re only open from like 5:30 to 2:30.”

Another participant shared that policies around take-home dosing added further restrictions on when they needed to get to the OTP: “I work, so it’s my fault, but if you get carries, you have to get there before like 11:45 in order to get your full however long you get carries for. And sometimes people don’t always make it at that time. What’s nice is that you’ll still get the dose that day, but then you just have to come back for the following day to get the rest of the take-homes.”

Some participants described that even when they did make it to the OTP, “sometimes it’s a really long wait” for their dosing visit. Another participant with a disability echoed the length and unpredictability of wait times, saying, “Sometimes you get there and there’s quite a crowd. You could sit and wait in line until your number comes up on the board to see the nurse a half hour, 40 minutes, maybe even an hour.”

While some participants were able to accommodate the OTP’s schedule and wait times, others found an added burden beyond time and effort it took to travel to the OTP. One participant described making multiple trips to the OTP each day during their work breaks, in hopes that wait times got shorter and allowed them to dose: “One of the reasons why I’m employed and I’ve been succeeding with my job is my punctuality. And with [OTP name], their sporadic times, wait time to dose would kind of interfere with that.… I was missing a few doses. And sometimes, I would go there and check in and wouldn’t be able to dose, and even come back on breaks and make a mad dash to go certain breaks and still wouldn’t be able to dose after going there twice in the day.”

### Theme 3: Dosing Visits, Employment, and Other Medical Care

Participants shared that even when they had a way to get to the OTP, there still were occasions when other factors in their life took precedence over a dosing visit. Sometimes participants felt the cost of travel was too high. One participant with a disability summarized this, saying, “I don’t have the money and the gas to get there all the time.” Another participant (who also had a disability) shared, “I spend $12 to $15 per fuel going there and back.” Survey responses echoed this, as per-visit costs averaged at $15.60 and ranged up to $200. Other times, participants experienced indirect costs when taking time to go to the OTP, such as from lost wages at work. “When I’m working, I was going every other day, so it was costing me a little bit more with that, because I couldn’t go,” said a participant. “I tried to get in early-early, and sometimes it would just last longer, so I’d have to be late to work, so that would cost a little bit more.”

Participants also expressed that competing life priorities—such as medical appointments to manage disabilities or other conditions, childcare, or “all kinds of things going on”—made regular travel to the OTP difficult. “I’ve always made it my biggest priority,” explained one participant. “But sometimes, you got to go to doctor appointment or a [social services] appointment or something, and you can’t get back and forth across town on the bus or something. It happens.”

Lastly, some participants perceived that regular dosing visits in the OTP increased the drug use they were exposed to. As one participant shared, “The more you go into the clinic, the more people you see that you know that still do this stuff, and that doesn’t help either.” Another described the burden of trying to maintain confidentiality in their workplace, saying, “I was working, I had a lot more shit going on in my life to where going once a week would have been more of a hardship if anything. So, going once a month was nice because I didn’t have to take weird time off work or explain to anybody what I was going to do or try and figure stuff out.”

### Theme 4: Risks for Return to Use and Stagnated Treatment and Recovery Progress

Participants shared that when they were unable to make it to the OTP for their dosing visit, that often left them feeling physically unwell. Participants found physical symptoms difficult and distressing to handle, sometimes leading to return to use. “I know early on, sometimes, because I was homeless at the time, it was hard to get into the clinic,” said a participant. “And sometimes that was the reason that I would slip and end up using, was because I couldn’t get into the clinic, and it was before the weekend or something. So, I would end up using fentanyl.”

Withdrawal symptoms from missed methadone doses also affected their ability to perform work or other life responsibilities. For some, return to use felt necessary to ensure they were able to get through the day: “On days that I missed, my performance at work was a really big—it’s really big to me. And then not feeling great because of my choices as far as withdrawing from opioids. I abused too, you know, [to] counteract my withdrawal symptoms.”

These experiences had negative consequences on participant’s feelings about themselves and their recovery. At times, these experiences also contributed to lost privileges including reduced take-home doses. Participants shared that this cycle of being unable to get to the OTP, missing a dose, and returning to use was counterproductive to their treatment and recovery goals. One participant summarized this, saying, “If they miss a dose, they might go right back to heroin. And that’s not gonna get anybody sober. That’s not helpful.”

## Discussion

This mixed-methods qualitative study found that many patients spent considerable time and money traveling to each dosing visit, while simultaneously managing housing instability, limited access to stable transportation, and disproportionate barriers to travel; 16.2% of our sample needed an assistive device for walking compared with 12.2% of the general US population.^29^ Qualitative data further illuminated that while distance itself was sometimes a barrier, OTP-related structural factors and/or conflicts with work, medical, or family obligations also mattered considerably. To date, most research that has explored transportation experiences for patients in methadone treatment have equated distance with difficulty.^15,30^ However, our data offer a more nuanced explanation for how patients may experience barriers across a spectrum of travel-related factors, including distance, disability, modality, life obligations, OTP operating structures, stigma, and financial costs. These findings shed light on the interlocking ways in which stigma against people who use drugs and structural ableism work in concert to disadvantage people seeking methadone treatment. It likewise speaks to the need for structural solutions that go beyond transportation support measures (eg, gas or transit fare reimbursement) and instead increase methadone availability (eg, location) and accessibility (eg, clinic hours, take-home flexibility).^31,32,33^

Since the start of its widespread use in 1972, methadone treatment has been an embodiment of the broad societal stigma for people who use drugs.^10,34,35,36^ As our data showed, people with OUD often face a continuous spiral of disruptions that perpetuate these structural disparities across all facets of their life, not just OUD treatment. For example, being able to work is both a facilitator of recovery and an important structural determinant that contributes to access to resources,^37^ like cars, but is in direct conflict with dosing visit requirements. OTPs have also been a target for drug dealers and drug sales, further contributing to risk for people seeking MOUD.^36^ The examples provided by participants in our study demonstrate how needing to maintain regular visits to the OTP for methadone dosing keeps patients trapped in cycles of limited economic mobility and treatment stagnation. However, data from other countries show that methadone can be safely delivered in low barrier outpatient settings in ways that may more readily promote the safety, well-being, and economic mobility of people seeking MOUD.^38,39,40^ The Modernizing Opioid Treatment Access (MOTA) Act,^41^ which proposes to expand methadone delivery via community-based pharmacies and addiction specialists outside of OTP settings, may be a pivotal catalyst in efforts to transform methadone delivery. Further expansion of mobile delivery services and novel mHealth tools like dosing directly observed via video^20^ will also be key strategies that can move the US away from what Krawczyk et al^42^ coin “methadone exceptionalism”—which siloes methadone as form of treatment needing to be controlled—and toward a practice that embodies accessible, patient-centered care.

### Limitations

Our study has several limitations. Our sample was drawn from a population of patients eligible for a video dosing pilot program specifically focused on reducing travel-related burden and may therefore not be representative of all patients in methadone treatment; however, these learnings still offer useful insights into the complex nature of barriers patients traveling to the OTP for dosing visits. Second, our qualitative data illuminated perspectives of predominantly White patients at a single nonprofit OTP in Washington state. More research is needed to understand how experiences differ across regions, OTP settings, patient populations (including those experiencing disabilities) beyond those we studied, and patient perspectives on potential solutions to increase methadone accessibility.

## Conclusions

In this qualitative study, we found that requiring regular visits for in-person observed methadone dosing involves disproportionate time, cost, and stress, which stagnated progress toward treatment and life goals. Data from this study continue to offer a compelling case for increasing the flexibility and patient-centeredness of methadone care delivery in the US.^10,43,44^

## Notes

Supplement. Data sharing statement
