# Supplementary material for: Patient Travel Experiences for Methadone Dosing Visits
Source: JAMA Netw Open. 2026 Oct 6;9(10):e2637601. doi: 10.1001/jamanetworkopen.2026.37601 (PMC13643578; doi:10.1001/jamanetworkopen.2026.37601)
Supplement: Supplementary file 1 [file jamanetwopen-e2637601-s001.pdf]

Data Sharing Statement

Austin. Patient Travel Experiences for Methadone Dosing Visits. *JAMA Netw Open*. Published October 06, 2026. doi:10.1001/jamanetworkopen.2026.37601

Data
Data available: No

Additional Information
Explanation for why data not available: Deidentified data may be shared upon request and approval from the study team.
